# Potential factors contributing to the poor antimicrobial efficacy of SAAP-148 in a rat wound infection model

**DOI:** 10.1186/s12941-019-0336-7

**Published:** 2019-12-03

**Authors:** Gabrielle S. Dijksteel, Magda M. W. Ulrich, Marcel Vlig, Peter H. Nibbering, Robert A. Cordfunke, Jan W. Drijfhout, Esther Middelkoop, Bouke K. H. L. Boekema

**Affiliations:** 1 Association of Dutch Burn Centres, Zeestraat 29, 1941 AJ Beverwijk, The Netherlands; 2 Dept. of Infectious Diseases, Leiden University Medical Centre, Albinusdreef 2, 2333 ZA Leiden, The Netherlands; 3 Dept. of Immunohematology and Blood Transfusion, Leiden University Medical Centre, Albinusdreef 2, 2333 ZA Leiden, The Netherlands; 4 Dept. of Plastic Reconstructive & Hand Surgery, Amsterdam University Medical Centres, Free University of Amsterdam, Amsterdam Movement Sciences, De Boelelaan 1117, 1081 HV Amsterdam, The Netherlands

**Keywords:** Antimicrobial peptides, SAAP-148, Efficacy, Topical therapy, Skin and soft tissue infections, Methicillin-resistant *Staphylococcus aureus*

## Abstract

**Background:**

We investigated the efficacy of a synthetic antimicrobial peptide SAAP-148, which was shown to be effective against Methicillin-resistant *Staphylococcus aureus* (MRSA) on tape-stripped mice skin. Unexpectedly, SAAP-148 was not effective against MRSA in our pilot study using rats with excision wounds. Therefore, we investigated factors that might have contributed to the poor efficacy of SAAP-148. Subsequently, we optimised the protocol and assessed the efficacy of SAAP-148 in an adapted rat study.

**Methods:**

We incubated 100 µL of SAAP-148 with 1 cm^2^ of a wound dressing for 1 h and determined the unabsorbed volume of peptide solution. Furthermore, 10^5^ colony forming units (CFU)/mL MRSA were exposed to increasing dosages of SAAP-148 in 50% (v/v) human plasma, eschar- or skin extract or PBS. After 30 min incubation, the number of viable bacteria was determined. Next, ex vivo skin models were inoculated with MRSA for 1 h and exposed to SAAP-148. Finally, excision wounds on the back of rats were inoculated with 10^7^ CFU MRSA overnight and treated with SAAP-148 for 4 h or 24 h. Subsequently, the number of viable bacteria was determined.

**Results:**

Contrary to Cuticell, Parafilm and Tegaderm film, < 20% of peptide solution was recovered after incubation with gauze, Mepilex border and Opsite Post-op. Furthermore, in plasma, eschar- or skin extract > 20-fold higher dosages of SAAP-148 were required to achieve a 2-log reduction (LR) of MRSA versus SAAP-148 in PBS. Exposure of ex vivo models to SAAP-148 for 24 h resulted in a 4-fold lower LR than a 1 h or 4 h exposure period. Additionally, SAAP-148 caused a 1.3-fold lower mean LR at a load of 10^7^ CFU compared to 10^5^ CFU MRSA. Moreover, exposure of ex vivo excision wound models to SAAP-148 resulted in a 1.5-fold lower LR than for tape-stripped skin. Finally, SAAP-148 failed to reduce the bacterial counts in an adapted rat study.

**Conclusions:**

Several factors, such as absorption of SAAP-148 by wound dressings, components within wound exudates, re-colonisation during the exposure of SAAP-148, and a high bacterial load may contribute to the poor antimicrobial effect of SAAP-148 against MRSA in the rat model.

## Background

Over the past decades, antibiotic resistance among bacteria has become a global public health problem. The acquisition of nosocomial Methicillin-resistant *Staphylococcus aureus* (MRSA) by injured patients not only increases the risk of sepsis but also delays wound healing [1–3]. Therefore, the need for alternative therapies that are effective and less likely to cause resistance has increased.

Recently, antimicrobial peptides (AMPs) are being studied as a potential alternative to antibiotics because of their anticipated favourable mechanism of action [4]. It has been demonstrated that AMPs — often small cationic peptides — eradicate a wide range of gram-negative and gram-positive bacteria by disrupting the bacterial membrane [5–7]. Initially, it was believed that AMPs secreted by human skin cells act as a chemical barrier that prevents the invasion of pathogens. However, AMPs display a number of additional functional activities besides their antimicrobial activity. They modulate several inflammatory responses, which in turn influence different cellular processes including wound healing [8–10]. Due to the promising efficacy profile of AMPs, several studies focus on the synthesis of naturally occurring AMPs and on modifications of these naturally occurring AMPs for more effective formulations for the topical treatment of infected or colonised wounds [11].

From a panel of synthetic AMPs, inspired on the structure of the human cathelicidin, LL-37, the synthetic peptide SAAP-148 was selected as the most promising AMP in reduction of bacterial counts [12]. This AMP was found effective against drug-resistant bacteria, persisters and biofilms. More interestingly, SAAP-148 effectively eradicated MSRA in an ex vivo human burn wound model (BWM) and an in vivo tape-stripped mouse model [12]. Subsequently, we studied the efficacy of SAAP-148 in an in vivo partial thickness wound model inoculated with MRSA in rats. Surprisingly, SAAP-148 was not effective in this model and therefore, several possible factors that may have contributed to the poor antimicrobial efficacy of SAAP-148 were identified and studied in in vitro and ex vivo experiments. Based on these experimental results, improvements to the protocol were implemented in a new rat study. The current paper reports on the findings of these in vitro, ex vivo and in vivo experiments.

## Methods

### Synthesis and formulation of SAAP-148

SAAP-148, inspired on LL-37 [13], was synthesised, purified and identified as described by Nell et al. [14]. Lyophilised SAAP-148 was dissolved and diluted in phosphate-buffered saline (PBS; Gibco, Paisley, UK). Aliquots of SAAP-148 in PBS were stored at − 20 °C until use. Hypromellose (HM) gels with or without SAAP-148 were prepared in two viscosities, i.e. 3:1 (3 parts pre-gel and 1 part peptide solution) and 3:10 (3 parts pre-gel and 10 parts peptide solution), as was previously described by Haisma et al. [15]. HM gels were stored at 4 °C. The SAAP-148 dosages used in the experiments were based on the optimal bactericidal effect of this peptide in the test condition.

### Tissue and blood plasma

Eschar is burned skin tissue that is debrided by a knife during surgery. Eschar and excess tissue that was collected after elective abdominal dermo-lipectomy were obtained under institutional guidelines and following the “code of conduct for responsible use”, drafted by Federa (Foundation Federation of Dutch Medical Scientific Societies) from the Red Cross Hospital (Beverwijk, the Netherlands). A dermatome (Aesculap AG & Co. KG, Tuttlingen, Germany) was used to prepare 0.8 mm thickness human skin grafts from the abdominal tissue. Rat (Wistar) and mouse (BL/6J) cadavers were purchased from the Amsterdam Animal Research Centre (Amsterdam, the Netherlands). A scalpel was used to collect full-thickness skin grafts from these animal cadavers. Grafts were stored for up to 2 weeks in Roswell Park Memorial Institute (RPMI) 1640 medium, supplemented with 2% (v/v) penicillin/streptomycin (P/S) (Gibco) at 4 °C. Prior to an experiment, the grafts were washed in RPMI 1640 medium at 4 °C for 24 h and subsequently in PBS for approximately 2 h to ensure the removal of P/S. Human blood plasma that was collected from three donors was obtained from Sanquin (Leiden, the Netherlands). The plasma was pooled, centrifuged at 2266 × *g* for 10 min, filtered using 0.2 µm filters to remove aggregates. Aliquots were stored at − 20 °C until use.

Eschar extracts were prepared by grinding 0.5 g eschar from three donors separately in 1 mL of PBS using a 7-mm metal bead and a TissueLyser LT (Qiagen, Venlo, the Netherlands) set at 50 Hz for 4 min. After centrifugation at 3600 × *g* for 5 min, the supernatants were pooled. The centrifugation step was repeated at 4200 ×* g* for 10 min and the clear eschar supernatant was stored in aliquots at − 20 °C. Skin extracts were prepared by cutting 0.5 g human skin from three donors into small pieces using a scalpel. Subsequently, the skin parts were grounded separately in 1 mL of PBS using the protein_01.01 program of the gentleMACS Dissociator (MACS Miltenyi Biotec, Bergisch Gladbac, Germany). After centrifugation at 3820 ×* g* for 15 min, the supernatants were filtered using 0.2 µm filters and pooled. This was stored in aliquots at − 20 °C.

### Absorption and/or adsorption of SAAP-148 by wound dressings

Approximately 1 cm^2^ of a wound dressing was added to a polypropylene vial containing 100 µL of SAAP-148 in PBS (76 nmol). After 1 h incubation at 37 °C and 5% CO_2_, the wound dressing was removed and the remaining volume of SAAP-148 containing solution was determined using a pipette. The following wound dressings were tested: gauze (BSN Medical GmbH, Hamburg, Germany), Mepilex border (Mölnlycke Health Care AB, Gothenburg, Sweden), Opsite Post-Op (Smith & Nephew Medical Limited, Hull, UK), Cuticell (BSN Medical GmbH) and Tegaderm film (3 M Health Care, Neuss, Germany). The recovered SAAP-148 containing solutions after incubation with a wound dressing were compared to the same solution without a wound dressing or with a low-adhesive material Parafilm (Merck, KGaA, Darmstadt, Germany), which was used in the in vivo study of de Breij et al. [12].

### Bacterial culture

A clinical isolate of MRSA, strain LUH14616, was stored in Luria–Bertani (LB) broth (Oxoid, Ltd, Basingstoke, UK) medium supplemented with 15% (v/v) glycerol at − 80 °C. Inoculae were grown on LB agar plates at 37 °C and 5% CO_2_ overnight. To create a mid-log phase growth culture, the bacteria were cultured in LB medium at 37 °C and 200 rpm for 4 h. After centrifugation at 3600 ×* g* for 5 min, the pellet was re-suspended in PBS to the desired bacterial concentration, estimated by using the optical density at 600 nm.

### Processing of samples to quantify viable bacteria

Samples were homogenised in a TissueLyser set at 50 Hz for 4 min using a 7-mm metal bead. During homogenisation of the samples, sodium polyanethol sulfonate (SPS; Merck KGaA) in PBS at a final concentration of 0.05% (wt/v) was used to neutralise SAAP-148 and prevent ongoing activity during processing [16]. For the neutralisation of 2% (wt/wt) mupirocin in an ointment (Bactroban; GlaxoSmithKline B.V., Zeist, the Netherlands) two times the manufacturer’s advised concentration of Dey-Engley broth (Merck KGaA) was used [17, 18]. Ten-fold serial dilutions of the homogenates were plated on LB agar plates for the in vitro studies, and MRSA*Select* agar plates (Bio-Rad, Lunteren, the Netherlands) for the animal studies to selectively identify MRSA from commensal bacteria. The number of viable bacteria — colony forming units (CFU) — was quantified after overnight incubation at 37 °C and 5% CO_2_. Results are expressed as the log10 reduction (LR), which was calculated by subtracting the log number of surviving bacteria after exposure to the peptide from the log number of bacteria in the negative control samples.

### In vitro killing assay

The bacterial suspension was diluted in PBS, human blood plasma, eschar extract or skin extract. 90 μL of the bacterial suspension were added to polypropylene vials containing 10 μL of SAAP-148 in HM gel, SAAP-148 in PBS, the empty HM gel or PBS. Final mixtures contained 50% (v/v) plasma, 50% (v/v) eschar extract, 50% (v/v) skin extract or PBS only and were briefly vortexed. After incubating the samples at 37 °C and 5% CO_2_ for 30 min, they were processed in 500 μL of PBS with drug-neutralisers to quantify the number of viable bacteria.

### Preparation of ex vivo models

Four types of ex vivo models were used: excision wound model, BWM, tape-stripped skin and intact skin. In brief, excision wound models were prepared with a dermatome (width 7 mm), which removed 0.3 mm of the upper part of the skin graft containing the epidermis. Subsequently, the graft was cut into pieces of 1 cm^2^ using a scalpel. BWMs were prepared by wounding 1 cm^2^ skin thermally with a soldering iron (10 × 2 mm) set at 95 °C for 10 s [19]. The tape-stripped skin was prepared by stripping the graft 20 times (replacing the tape after each time) using Tensoplast (BSN Medical GmbH) [12]. Afterward, the tape-stripped graft was cut into pieces of 1 cm^2^. Intact skin models were prepared by cutting the skin graft into pieces of 1 cm^2^.

### Infection and treatment of ex vivo skin

Ex vivo models were inoculated with 10 µL of MRSA and treated with SAAP-148 in HM gel, SAAP-148 in PBS, wound dressings containing SAAP-148, the empty HM gel or PBS at 37 °C and 5% CO_2_. The samples were processed in 1 mL of PBS with drug-neutralisers to quantify the number of viable bacteria.

### Efficacy of SAAP-148 in a rat model

Immune competent male and female rats (Wistar) of 8 to 10 weeks old with a minimum weight of 160 g were purchased from Envigo (Horst, the Netherlands). These animals were kept under specific pathogen-free conditions and were housed in individually ventilated cages. Wood-shavings were used as bedding material and long paper strips as enrichment. The rats were provided with tap water and an irradiated-sterilised pelleted diet ad libitum.

The sample size was calculated with data of in vitro studies and literature [20]. The type I error probability was set at 0.05. To identify a 140-fold decrease in bacterial counts and assuming a standard deviation (SD) of 80, a sample size of 5 per group (with a power of 0.8) was required. Considering the possibility of ˂ 10% drop-out, a sample size of 6 per group per treatment time was used. To minimize the number of experimental animals two wounds, one on each flank, were prepared on the back of the rats. Six groups, each with one wound on 6 male and 6 female rats were used.

The MRSA inoculated wounds were treated with 306 (group 1) or 612 nmol (group 2) SAAP-148 in 3:10 HM gel, 153 (group 3) or 306 nmol (group 4) SAAP-148 in PBS, the empty HM gel (group 5) or 2% (wt/wt) Bactroban (group 6) (see Additional file 1: Table S1 for SAAP-148 dosages in percentages). A lower dosage of SAAP-148 in PBS was used to prevent possible toxicity effects caused by the instantaneous high availability of the peptide in the wounds. Four h or 24 h after treatment, six wounds (i.e. one wound on 3 male and 3 female rats) of each group were sampled.

Thirty to sixty minutes pre-operative, 36 rats were subcutaneously injected with analgesic Temgesic (1 µg/100 g) and were given tap water containing Temgesic (0.01 mg/kg) for 24 h to reduce post-operative pain. Additionally, 1–2 h pre-operatively the rats were subcutaneously injected with Carprofen (5 mg/kg) to reduce pain and fever. During the interventions, the rats were kept under anesthesia with 2% isoflurane in oxygen. To maintain the body temperature a heated pad was used. The back of the rats was shaved using an electric razor and the remaining hair was removed using Veet hair removal cream for sensitive skin (RB Health Care, Hoofddorp, the Netherlands). The skin was disinfected with 70% alcohol and sprayed with analgesic 1% Lidocaine to locally anesthetize the skin. Two excision wounds of approximately 1 cm^2^ were created on the back of the rats with a dermatome set at 0.8 mm. To confirm sterility, the wounds were swabbed using forensic swabs (Sarstedt AG & Co, Nümbredt, Germany) soaked in sterile PBS. Subsequently, the wounds were inoculated by placing gauze containing 100 µL of 10^8^ CFU/mL MRSA on the wound, which was kept in place with a self-adhesive wound dressing Petflex (3 M Health Care) and a Leukosilk plaster (BSN Medical GmbH) around the entire trunk. Post-operative, the rats received a subcutaneous injection with analgesic Metacam (1 mg/100 g) to further reduce pain. After overnight inoculation, the wounds were wiped three times using a PBS-moist gauze, similar to human clinical protocols. Then the wounds were swabbed to determine the starting bacterial load. They were topically treated with 100 µL of 306 or 612 nmol SAAP-148 in 3:10 HM gel, 153 or 306 nmol SAAP-148 in PBS, the empty HM gel (negative control) or 2% (wt/wt) Bactroban (positive control). The wounds were covered with Tegaderm film followed by Petflex and a Leukosilk plaster. After 4 h and 24 h treatment, the rats were euthanised using saturated CO_2_/O_2_ followed by CO_2_ only. Subsequently, the number of superficially located bacteria in the wounds were determined using swabs and those within the tissue were determined using 4 mm punch biopsies. Samples were processed in 1 mL of PBS with drug-neutralisers to determine the number of surviving bacteria.

### Statistical analysis

To determine the statistically significant differences between two sample groups the non-parametric Kruskal–Wallis test and the Mann–Whitney rank sum test were used. For comparison of two time points, the Wilcoxon signed-rank test was used. p-values ≤ 0.05 were considered statistically significant.

## Results

In a previous experiment, SAAP-148 in 3:1 HM gel failed to cause a significant LR, despite repetitive daily administration of SAAP-148 to these wounds (Additional file 1: Figure S1). Therefore, we performed additional in vitro and ex vivo experiments to determine some of the possible factors contributing to this poor antimicrobial effect of SAAP-148, such as formulation in HM gel, absorption and/or adsorption of SAAP-148 by wound dressings, exposure periods of SAAP-148, bacterial loads and components in the wound micro-environment. Subsequently, we optimised the in vivo protocol and assessed the efficacy of SAAP-148 in an adapted rat study.

### The viscosity of HM gel affects the bactericidal efficacy of SAAP-148

**Fig. 1:**
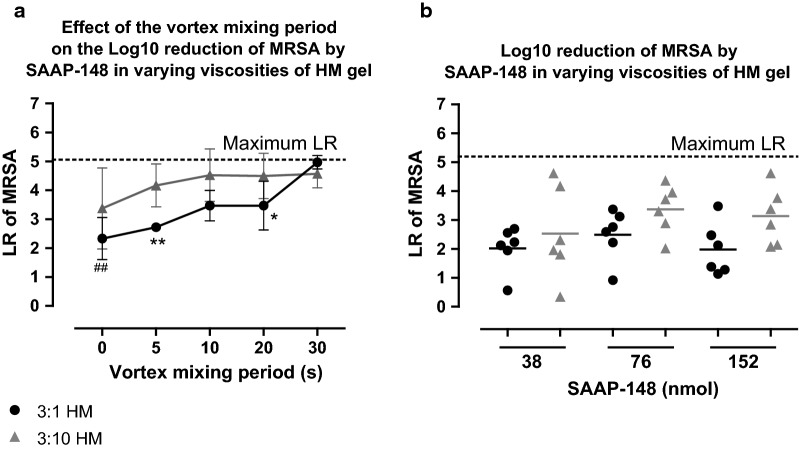
**Efficacy of SAAP-148 formulated in HM gels.** **a** In vitro killing of 10^5^ CFU/mL MRSA by 7.6 nmol SAAP-148 in 3:1 or 3:10 HM gel or the empty HM gel as negative control. The mixtures were either vortexed or not. The vortex mixing period is indicated on the x-axis. The mean ± SD of six experiments performed in duplicate is shown. ^#^ indicates significant difference compared to the LR at 30 s (^##^p < 0.01) and * indicates significant difference (*p < 0.05; **p < 0.01) compared to SAAP-148 in 3:10 HM. **b** The ex vivo efficacy of SAAP-148 in 3:1 or 3:10 HM gels. Excision wound models were inoculated with approximately 10^5^ CFU for 4 h and exposed to 50 µL of 3:1 or 3:10 HM gels containing 38 nmol, 76 nmol, 152 nmol SAAP-148 or the empty HM gel (negative control) for 1 h. The mean of six independent experiments performed in triplicate is shown. Results are expressed as the LR

To exclude the possibility that SAAP-148 was unable to exert its antimicrobial effect because it remained in the viscous 3:1 HM gel and did not reach the bacteria in the wound, we explored an alternative viscosity of the HM gel to potentially improve the release of the peptide from the HM gel and thus its bactericidal effect. We assessed the effect of vortex mixing on the efficacy of SAAP-148 (7.6 nmol) in 3:1 or 3:10 HM gel using in vitro killing assays. The results revealed that increasing the vortex mixing period from 0 to 30 s resulted in a 2-fold (p < 0.01) higher LR for SAAP-148 in 3:1 HM, whereas for SAAP-148 in 3:10 HM gel the LR at 0 s was already high and comparable to the LR at 30 s. Additionally, the LR caused by SAAP-148 in 3:10 HM gel was 1.5 (p < 0.01) and 1.3-fold (p < 0.05) higher than for SAAP-148 in 3:1 HM gel at vortex mixing periods of 5 s and 20 s, respectively (Fig. 1a).

Furthermore, we compared the efficacy of SAAP-148 in 3:1 and 3:10 HM gel using ex vivo excision wound models. The ex vivo models were inoculated for 4 h with approximately 10^5^ CFU/mL MRSA and exposed to 50 µL of SAAP-148 in 3:1 or 3:10 HM gels or the empty HM gel for 1 h. Although we could not establish significant differences in the bactericidal efficacy of SAAP-148 in 3:10 versus 3:1 HM gel, the LR after exposure to SAAP-148 in 3:10 HM gel seemed greater, especially at high dosages (Fig. 1b).

Overall, SAAP-148 in the 3:1 HM gel caused a lower LR as compared to SAAP-148 in the 3:10 HM gel. Therefore, the following experiments were performed with SAAP-148 in 3:10 HM gel or in PBS to assure the most optimal availability of the peptide to interact with bacteria.

### Effect of wound dressings on the efficacy of SAAP-148

**Fig. 2:**
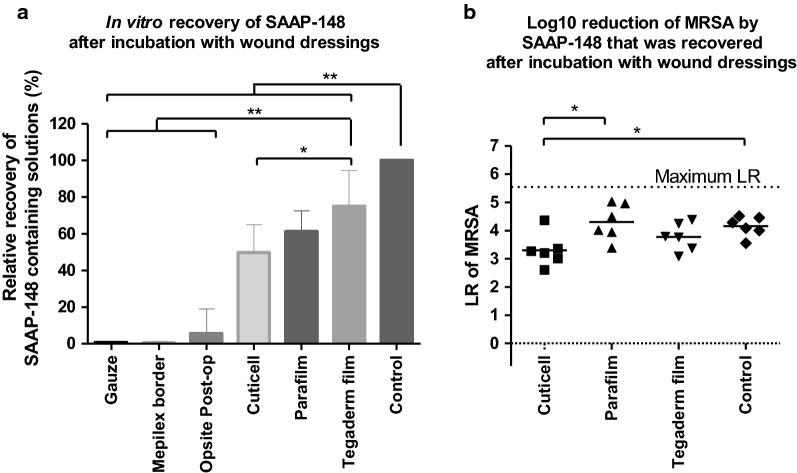
**Absorption and/or adsorption by wound dressings and its effect on the efficacy of SAAP-148.** **a** 100 µL of SAAP-148 in PBS (76 nmol) were incubated with or without 1 cm^2^ of a wound dressing or low adhesive Parafilm for 1 h. Subsequently, the remaining volume of SAAP-148 containing solutions were determined. Results are expressed as the relative recovery of the solutions in percentages of control samples without a wound dressing. Data are shown as mean ± SD of six independent experiments. **b** In vitro killing of MRSA by 10 µL of SAAP-148 containing solutions that were remaining after incubation with or without Cuticell, Parafilm and Tegaderm film. PBS was used as negative control. Results are expressed as the LR. The mean of six independent experiments performed in triplicate is shown. * indicates significant difference (*p < 0.05; **p <0.01) compared to control samples

In the pilot rat study gauze and Cuticell were used as wound dressings for the excision wounds (Additional file 1: Information). Absorption and/or adsorption of the SAAP-148 treatment by gauze and Cuticell might have contributed to the poor antimicrobial efficacy of SAAP-148 in vivo. Therefore, we determined the amount of peptide solution that could be recovered after 1 h incubation of 100 µL of SAAP-148 in PBS (76 nmol) with 1 cm^2^ of different wound dressings or a low adhesive material Parafilm. Compared to the control samples without a wound dressing, < 20% of the peptide solution was recovered after incubation with gauze, Mepilex border or Opsite Post-Op. In contrast, 45%, 56% and 75% of the peptide solution were recovered after incubation with Cuticell, Parafilm or Tegaderm film, respectively (Fig. 2a). Therefore, we then investigated the in vitro efficacy of only the SAAP-148 solutions that were recovered after incubation with Cuticell, Parafilm or Tegaderm film and determined if the presence of these wound dressings affected the antimicrobial activity of SAAP-148 against MRSA. Ten µL of the recovered SAAP-148 solution that had been incubated in the presence of Cuticell caused a 1.3-fold (p < 0.05) lower mean LR than SAAP-148 that was not incubated in the presence of a wound dressing (positive control) or SAAP-148 that was incubated in the presence of Parafilm (Fig. 2b). Thus, the presence of Cuticell, which is a moist silicon wound contact layer, negatively affected the antimicrobial activity of SAAP-148. On the contrary, the presence of Parafilm or Tegaderm film in the peptide solutions did not have a significant effect on the antimicrobial activity of SAAP-148 against MRSA.

**Fig. 3:**
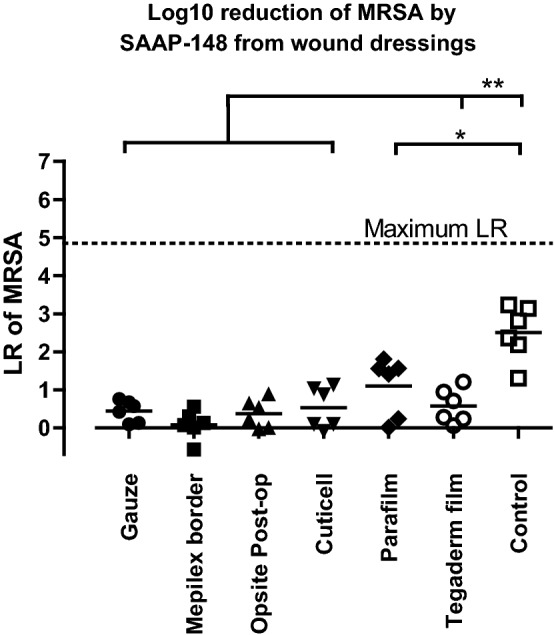
**Efficacy of SAAP-148 from a wound dressing.** Ex vivo excision wound models were inoculated with approximately 10^5^ CFU/mL MRSA for 1 h and exposed to a wound dressing containing 15 nmol SAAP-148 or with 20 µL of PBS for 1 h. Results are expressed as the LR. The mean of six independent experiments performed in triplicate is shown. * indicates significant difference (*p < 0.05; **p < 0.01) as compared to control samples

Next, we evaluated whether SAAP-148 that was absorbed and/or adsorbed by the different wound dressings or Parafilm could still be released to eradicate bacteria. Ex vivo excision wound models were inoculated with 10^5^ CFU/mL MRSA for 1 h and exposed to a wound dressing containing 15 nmol SAAP-148, 20 µL of SAAP-148 in PBS (15 nmol) (positive control) or PBS (negative control) for 1 h. Results showed that all the tested wound dressings containing SAAP-148 caused a mean LR that was 2.3-fold (p < 0.05) or > 4-fold (p < 0.01) lower than for SAAP-148 in PBS (positive control) (Fig. 3). This suggests that SAAP-148 was not sufficiently released from these wound dressings to eradicate MRSA on ex vivo skin.

In summary, Tegaderm film is a more appropriate wound dressing than gauze or Cuticell because it is less likely to absorb the peptide or affect its antimicrobial activity.

### Exposure of MRSA infected skin models to SAAP-148 for 1 h, 4 h or 24 h

**Fig. 4:**
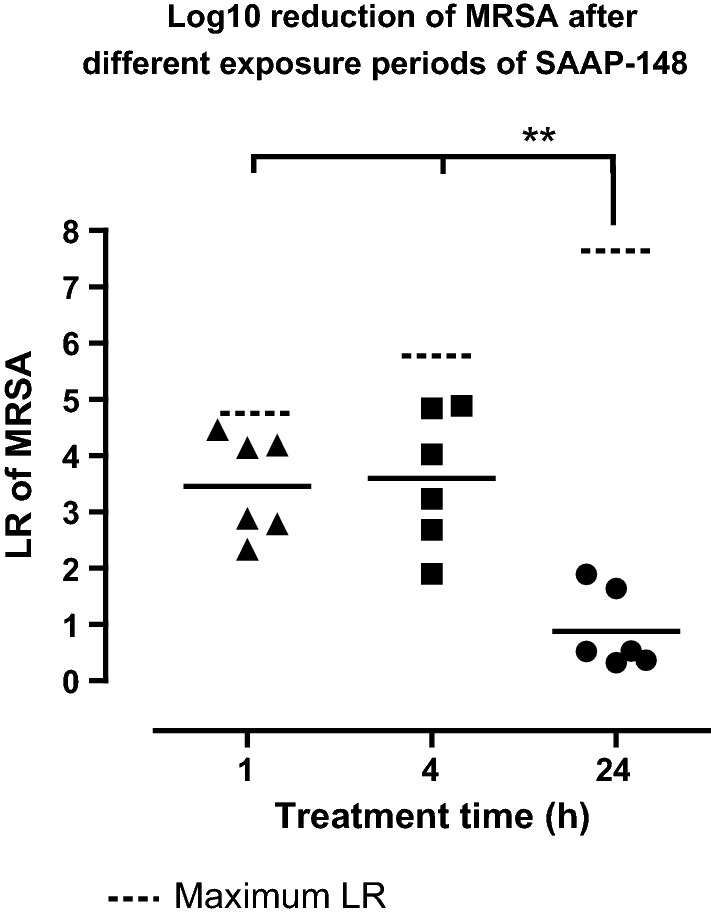
**The effectiveness of SAAP-148 over time.** Ex vivo excision wound models were inoculated with approximately 10^5^ CFU/mL MRSA for 1 h and subsequently exposed to 60 nmol SAAP-148 in 20 µL of PBS for 1 h, 4 h, or 24 h. PBS was used as negative control. Results are expressed as the LR. Data represent the mean of six independent experiments performed in triplicate. * indicates significant difference (*p < 0.05; **p < 0.01) as compared to the LR at a 1 h or 4 h exposure period of SAAP-148

To establish the efficacy of SAAP-148 over time, ex vivo excision wound models were inoculated with 10^5^ CFU MRSA for 1 h and exposed to 20 µL of SAAP-148 in PBS (60 nmol) or PBS (negative control). After 1 h, 4 h or 24 h, the mean LR caused by SAAP-148 was determined. Exposure of the models to SAAP-148 for 1 h or 4 h resulted in a LR of 3.5 and 3.6, respectively. However, exposure of the models to SAAP-148 for 24 h resulted in a mean LR of only 0.9, which was significantly (p < 0.01) lower than the LR caused by SAAP-148 after a 1 h or 4 h exposure period (Fig. 4). Thus, SAAP-148 is effective against MRSA after 1 h or 4 h but not after 24 h, indicating that the peptide should be applied multiple times a day.

### SAAP-148’s efficacy in relation to bacterial load

**Fig. 5:**
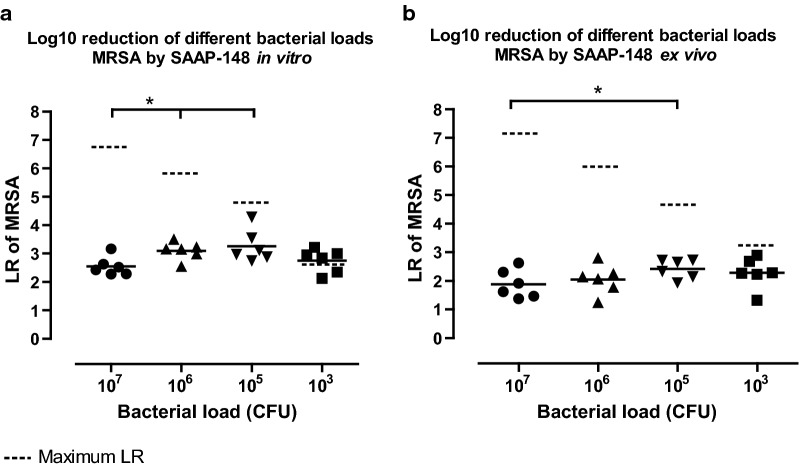
**SAAP-148 against different bacterial loads of MRSA.** The effect of 7.6 nmol SAAP-148 in PBS on different bacterial loads. **a** In vitro killing of 10^3^, 10^5^, 10^6^ or 10^7^ CFU MRSA by 10 µL of PBS or SAAP-148 in PBS after 30 min incubation. **b** Ex vivo excision wounds were inoculated with 10^3^, 10^5^, 10^6^ or 10^7^ CFU MRSA for 1 h and exposed to 10 µL of SAAP-148 in PBS or PBS for 1 h. Results are expressed as the LR. Data represent the mean of six independent experiments performed in triplicate. * indicates significant difference (*p < 0.05; **p < 0.01) as compared to the LR caused by SAAP-148 at 10^7^ CFU MRSA

The relatively high bacterial load (10^7^ CFU/wound) in the excision wounds of the rat model might have contributed to the poor antimicrobial effect of SAAP-148 in the pilot study (Additional file 1: Information). To evaluate this, in vitro killing assays were performed where 10^3^, 10^5^, 10^6^ or 10^7^ CFU MRSA were exposed to 10 µL of SAAP-148 in PBS (7.6 nmol) or PBS (negative control). SAAP-148 eradicated an absolute higher number of bacteria as the bacterial load increased from 10^3^ CFU to 10^7^ CFU. However, SAAP-148 caused a 1.3-fold (p < 0.05) lower mean LR at a bacterial load of 10^7^ CFU than at 10^6^ or 10^5^ CFU MRSA (Fig. 5a). The same was found for ex vivo excision wound models (Fig. 5b). Notably, the mean LR caused by SAAP-148 was 1.3 to 1.5-fold (p < 0.01) lower in the presence of ex vivo skin at a bacterial load of 10^6^ or 10^5^ CFU MRSA. Thus, SAAP-148 is less effective against high bacterial loads of 10^7^ CFU MRSA, which was used in the in vivo rat study.

### In vitro activity of SAAP-148 in plasma, eschar or skin extract

**Fig. 6:**
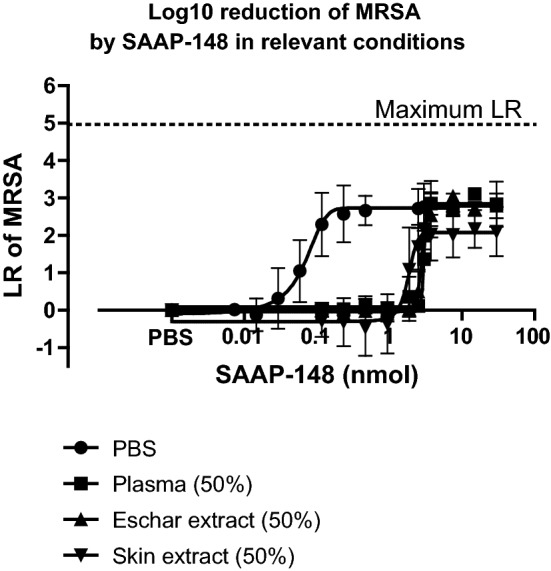
**In vitro killing assay of SAAP-148 against MRSA in PBS, plasma, eschar or skin extract.** Approximately 10^5^ CFU/mL MRSA that was suspended in PBS or in PBS with 50% (v/v) plasma, or 50% (v/v) eschar extract, or 50% (v/v) skin extract, was exposed to 10 µL of increasing dosages (0–30 nmol) of SAAP-148 in PBS. Results are expressed as the LR versus the amount of SAAP-148 in nmol. Each value represents the mean ± SD of three independent experiments performed in duplicate

To determine the efficacy of SAAP-148 in biologically relevant conditions, we performed additional in vitro killing assays in PBS with 50% (v/v) plasma, 50% (v/v) eschar extract or 50% (v/v) skin extract. In these conditions, SAAP-148 eradicated MRSA in a dose-dependent manner. However, higher dosages of SAAP-148 were required to achieve a 2-LR in 50% (v/v) plasma, 50% (v/v) eschar extract or 50% (v/v) skin extract than in PBS, i.e.: 2.3 nmol, 3.1 nmol, 2.8 nmol and 0.1 nmol, respectively. Strikingly, even at the highest peptide dosage of 30 nmol, still approximately 10^2^ CFU/mL MRSA survived in all of these conditions (Fig. 6).

### Efficacy of SAAP-148 against MRSA in different skin models

Because the earlier animal experiments of SAAP-148 were performed using a tape stripped mouse model [12], we evaluated the efficacy of SAAP-148 in different environments using ex vivo skin models. The models were inoculated with approximately 10^5^ CFU/mL MRSA and after 1 h they were exposed to 20 µL of SAAP-148 in PBS (60 nmol) or PBS (negative control) for 1 h.

**Fig. 7:**
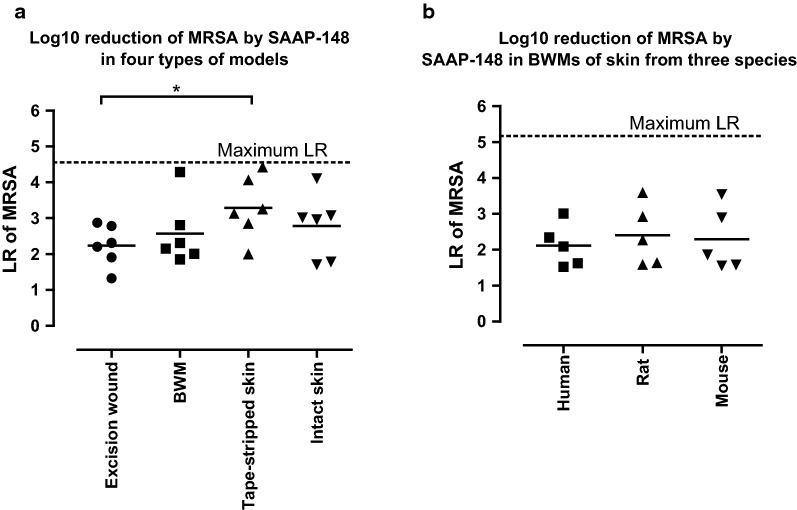
**Antimicrobial activity of SAAP-148 against MRSA in different ex vivo** **models.** Ex vivo models were inoculated with approximately 10^5^ CFU/mL MRSA for 1 h and then exposed to 20 µL of PBS with or without 60 nmol SAAP-148 in PBS for 1 h. **a** The efficacy of SAAP-148 in four wound types, i.e. excision wounds, BWM, tape-stripped skin and intact. **b** The efficacy of SAAP-148 in BWMs of skin from three species, i.e. human, rat and mouse. Results are expressed as the LR. Data represent the mean of at least five independent experiments performed in triplicate. * indicates significant difference (*p < 0.05; **p < 0.01)

First, we evaluated the efficacy of SAAP-148 in four types of ex vivo models prepared from human skin, i.e. the excision wound model, BWM, tape-stripped skin and intact skin. The LR caused by SAAP-148 varied among the different models. However, SAAP-148 caused a 1.5-fold lower (p < 0.05) mean LR in the excision wound models than on tape-stripped skin (Fig. 7a).

Next, we determined the efficacy of SAAP-148 using ex vivo BWMs prepared from skin of three species, i.e. human, rat and mouse. SAAP-148 caused a comparable mean LR in the BWMs of these different species (Fig. 7b). Significant differences were not noted.

Thus, SAAP-148 is less effective against MRSA in excision wound models than on tape-stripped skin and its potency is independent of the skin characteristics from the tested species.

### SAAP-148 treatment in a rat model

**Fig. 8:**
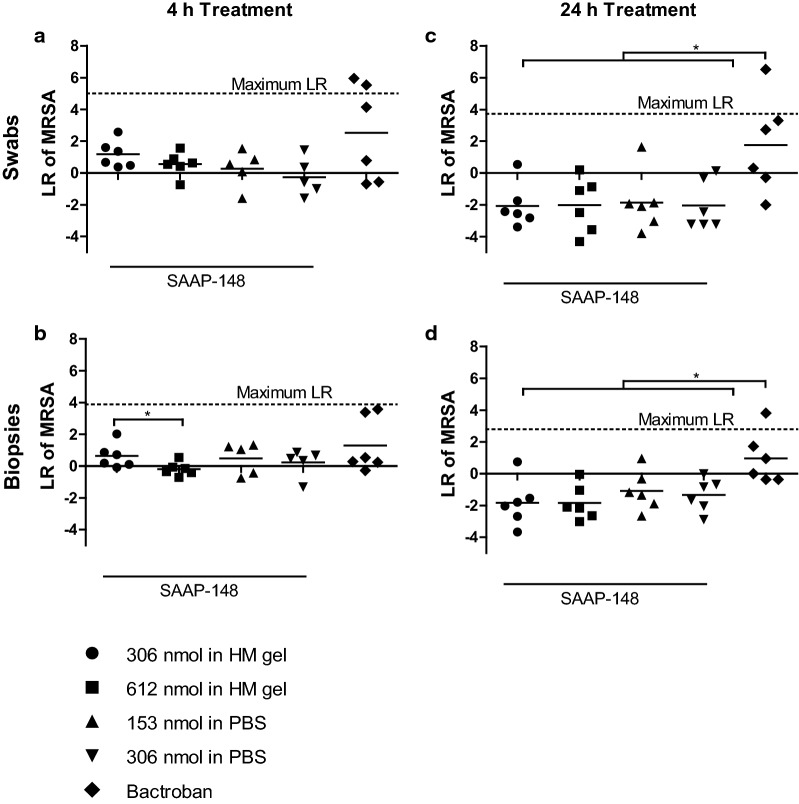
**Efficacy of SAAP-148 in a rat model.** The bacterial load of excision wounds from rats was determined using swabs **(A/C)** and punch biopsies (**b**, **d**) of the wounds. After an overnight inoculation with MRSA, the wounds were treated for 4 h (**a**, **b**) or 24 h (**c**, **d**) with 100 µL of 3:10 HM gel containing 306 or 612 nmol SAAP-148 or PBS containing 153 or 306 nmol SAAP-148 or the empty HM gel (negative control) or 2% (wt/wt) Bactroban (positive control). Results are expressed as the LR. Data represent the mean of at least five samples. * indicates significant difference (*p < 0.05; **p < 0.01)

Based on the results above, we adapted the protocol for the study of the efficacy of SAAP-148 in MRSA-inoculated excision wounds in rats. The wounds on the back of 36 rats were inoculated with 100 µL of 10^8^ CFU/mL MRSA and after 24 h (immediately before the treatment), the wound-swabs showed bacterial counts of approximately 10^6^ CFU/wound. The wounds were topically treated with 100 µL of 306 or 612 nmol SAAP-148 in 3:10 HM gel, 153 or 306 nmol SAAP-148 in PBS, the empty HM gel (negative control) or 2% (wt/wt) Bactroban (positive control). Treatment with SAAP-148 or Bactroban for 4 h or 24 h did not eradicate a significant number of MRSA as compared to the negative control. Also, the mean bacterial counts of all SAAP-148-treated wounds were > 2-fold (p < 0.05) higher than that of the negative- and positive control after 24 h treatment, resulting in a negative LR in Fig. 8c, d.

## Discussion

The aim of the current study is to investigate the poor antimicrobial effect of a novel AMP, SAAP-148, against MRSA in excision wounds in vivo. In our pilot rat study (Additional file 1: Figure S1), SAAP-148 failed to reduce the bacterial counts in the wounds, which was an unexpected finding in view of the previous in vitro and in vivo studies with SAAP-148 [12]. Therefore, we focused on identifying some of the potential factors contributing to the poor antimicrobial effect of SAAP-148 in this model. We tested the HM gel formulation, absorption and/or adsorption of SAAP-148 by wound dressings, exposure periods of SAAP-148, bacterial loads and components in the wound micro-environment. Most of the tested aspects negatively influenced the bactericidal effect of SAAP-148 to varying degrees and the conditions with the most optimal bactericidal effect of SAAP-148 were used in the adapted rat study.

Despite all the adjustments to improve the in vivo protocol and the performance of SAAP-148, this peptide failed to cause a significant LR as compared to the negative control (HM gel) (Fig. 8). Absorption by the wound dressing, gauze, was probably a major factor contributing to the poor antimicrobial effect of SAAP-148 in the pilot rat study but not in the adapted rat study, where the non-absorbing Tegaderm film was used. In this study, the wound environment played the major part in the failure of SAAP-148. We found that at least 20-fold higher dosages of SAAP-148 were required to eradicate MRSA effectively, especially in the presence of ex vivo skin (Fig. 5), 50% (v/v) plasma, 50% (v/v) eschar extract or 50% (v/v) skin extract (Fig. 6). Previously, a similar impact of a protein-rich environment on the efficacy of antimicrobial agents was reported. The presence of proteases, human dentin, plasma proteins including albumin and lipoproteins such as high-density lipoprotein, and lipopolysaccharide inhibited the antimicrobial activity of the tested antimicrobial agents [12, 21–25]. This suggests a reduced availability of antimicrobials to interact with bacteria as a result of protein binding and/or proteolytic degradation. It complicates the therapeutic application of SAAP-148 for excision wounds, which consist of a protein-rich environment.

Moreover, increasing the peptide dosages did not result in the complete elimination of the bacteria in the adapted rat study but also not in the in vitro and ex vivo experiments, which could be explained by *i)* the presence of bacterial aggregates that may form biofilms and *ii)* persistent bacteria as described by Bay et al. [26]. This is in contrasts to the findings of de Breij et al. who reported that SAAP-148 is effective against biofilms and eradicates persistent bacteria [12]. The differences in results might be due to the higher susceptibility of the *S. aureus* strain JAR060131 or due to limited protein presence in the tape-stripped mouse model that was used in their study. To elaborate, SAAP-148 eradicated a comparable number of bacteria in ex vivo models with skin from different species (Fig. 7b) but SAAP-148 caused a 1.5-fold lower (p < 0.05) mean LR in the excision wounds than on tape-stripped skin (Fig. 7a). Taking into consideration that deep acute wounds more likely consist of a protein-rich environment (plasma and exudate), the efficacy differences of SAAP-148 between superficial and partial thickness acute wounds might be even larger clinically. Of note, the absence of a drug-neutraliser for SAAP-148 after sampling in the study of de Breij et al. could have resulted in an extended exposure of SAAP-148 to the bacteria. This could have resulted in the eradication of surviving bacteria after sampling the wounds and thus only an apparent effective eradication of bacteria in the wound [16].

The need for high treatment dosages to completely eliminate bacteria in vivo has been emphasised by Tsai et al. who topically exposed bacteria to > 1000 times the minimum inhibitory concentration of gentamicin or minocycline to assure an effective bacterial elimination [27]. However, high antimicrobial dosages may result in adverse effects. Alternatively, optimising the peptide formulation to cause a burst release upon the presence of bacterial proteins, could result in a selective and complete elimination of bacteria. Such a phenomenon was previously shown for Prodrugs, whereby the active AMP is released upon degradation of the protease-sensitive peptide, which was bound to the positively charged group of the AMP for protection [28]. Enzymes, such as beta-lactamase can break down the protecting peptide, which in turn results in high availability of the active AMP to eradicate bacteria completely [28].

The importance of eradicating bacteria completely is supported by the results obtained from our ex vivo experiment using excision wound models and different exposure periods of SAAP-148 (Fig. 4). The results indicated that SAAP-148 is effective within the first few hours but that bacteria that survived are able to proliferate and re-colonise the wounds. This might have been the case in our pilot rat study, where the efficacy of SAAP-148 was only studied after 24 h treatment (Additional file 1: Information). Accordingly, Müsken et al. reported that the time required for surviving bacteria to repopulate the biofilm could be taken as a measure for the effectiveness of the antimicrobial treatment and that this is also dependent on the duration of antibiotic treatment and interval of drug administration [29]. This suggests that repeated dosing of SAAP-148 would be required after short treatment times to effectively eradicate bacteria. However, a short treatment time of 1 h or 4 h is not preferable in clinical settings because of the high workload for medical personnel but also for the burden to the patient [30, 31]. Possibly, a continuous and sustained release of the peptide during a 24 h treatment could eradicate bacteria effectively without causing adverse effects. Previously, such functionalised wound dressings have been prepared for several antimicrobial agents and have shown to be effective [32–34]. However, MRSA was not effectively eradicated in our ex vivo experiment, where colonised ex vivo excision wounds had been exposed to wound dressings containing SAAP-148. This suggests that SAAP-148 was not sufficiently released from the wound dressings to eradicate bacteria (Fig. 3). Hence, more work focussing on the release profile is needed to develop a functionalised wound dressing for SAAP-148.

## Conclusions

We found several factors that could have contributed to the poor antimicrobial effect of SAAP-148 in the pilot rat study: (i) the slow release profile of SAAP-148 from the 3:1 HM gel, (ii) the absorption of SAAP-148 by gauze and subsequently an incomplete release of the peptide from the wound dressing, (iii) the reduced bactericidal efficacy of SAAP-148 caused by the presence of the moist silicon wound contact layer Cuticell, and (iv) re-colonisation during the prolonged treatment time of 24 h. After performing the adapted rat study with SAAP-148, we concluded that the main factors contributing to the poor antimicrobial effect of SAAP-148 were related to the wound micro-environment, i.e. components within the wound exudates and the relatively high bacterial load in the wounds.

## Supplementary information

**Additional file 1.** SAAP-148 dosages to eradicate bacteria.

## Data Availability

The datasets used and analyzed during the current study are available from the corresponding author on reasonable request.

## Abbreviations

AMPs: antimicrobial peptides
BWM: burn wound model
CFU: colony forming units
HM: hypromellose
LR: log reduction
LB: Luria–Bertani
MRSA: methicillin-resistant *Staphylococcus aureus*
PBS: phosphate-buffered saline
RPMI: Roswell Park Memorial Institute
SD: standard deviation
SPS: sodium polyanethol sulfonate
P/S: penicillin/streptomycin
