# Supplementary material for: Potential factors contributing to the poor antimicrobial efficacy of SAAP-148 in a rat wound infection model
Source: Ann Clin Microbiol Antimicrob. 2019 Dec 3;18:38. doi: 10.1186/s12941-019-0336-7 (PMC6891976; doi:10.1186/s12941-019-0336-7)
Supplement: Supplementary file 1 — Additional file 1. SAAP-148 dosages to eradicate bacteria. [file 12941_2019_336_MOESM1_ESM.docx]

**Potential factors contributing to the poor antimicrobial efficacy of SAAP-148 in a rat wound infection model**

**Authors:** Gabrielle S Dijksteel,^⁕1,4^ Magda MW Ulrich,^1,4^ Marcel Vlig,^1^ Peter H Nibbering,^2^ Robert A Cordfunke,^3^ Jan W Drijfhout,^3^ Esther Middelkoop,^1, 4^ and Bouke KHL Boekema^1^

**Additional file 1: SAAP-148 dosages to eradicate bacteria.**

Information: Pilot rat study for the determination of SAAP-148’s efficacy.

Figure S1: Efficacy of SAAP-148 against MRSA or *Pseudomonas aeruginosa*

Table S1: Conversion of SAAP-148 dosages.**Additional file 1: SAAP-148 dosages to eradicate bacteria**

**Information: Pilot rat study for the determination of SAAP-148’s efficacy.**

The efficacy of SAAP-148 in 3:1 HM gel was assessed in a pilot rat study, which was similar to the experiment described in the manuscript. The experimental procedure is mentioned in **Methods: Efficacy of SAAP-148 in a rat model** of the manuscript.

After an acclimatization period of 14 days, two wounds were prepared on the back of 48 rats. Both wounds of the rats from group 3 and 4 were inoculated with an MRSA stain LUH14616. For the other groups, the wounds on the left flank were inoculated with MRSA and those on the right flank were inoculated with a *Pseudomonas aeruginosa* (PA) strain PAO1. After overnight inoculation with approximately 10^7^ CFU MRSA or PA, the wounds were treated with 15, 30 or 60 nmol SAAP-148 in 3:1 HM gel, the empty 3:1 HM gel or 2% (wt/wt) Prontosan gel (B Braun Medical B.V., Oss, the Netherlands) on day 1, 2, 3 and 4 (see table below). On day 3 and 7, 24 rats were euthanised to determine the bacterial counts using swabs and 4 mm punch biopsies.

| **Experimental groups** | **Group 1/2** | **Group 3/4** | **Group 5/6** | **Group 7/8** |
| --- | --- | --- | --- | --- |
| **Inoculation** | MRSA – PA | MRSA – MRSA | MRSA – PA | MRSA – PA |
| **Treatment** | 60 nmol  SAAP-148 in 3:1 HM gel | 15 – 30 nmol  SAAP-148 in 3:1 HM gel | 3:1 HM gel | 2% (wt/wt) Prontosan gel |

**Figure S1: Efficacy of SAAP-148 against MRSA or *Pseudomonas aeruginosa***

After overnight inoculation, the bacterial load of the wounds was determined using swabs **(A/C)** before the daily treatment with 20 µL of 3:1 HM gels containing 15 nmol, 30 nmol, 60 nmol SAAP-148 or the empty HM gel (negative control) or 2% (wt/wt) Prontosan gel (positive control). Additionally, punch biopsies **(B/D)** of the wounds were collected to determine the bacterial load on day 3 and day 7. Results represent the mean of at least five samples that were treated with 60 nmol SAAP-148 in 3:1 HM gel or Prontosan gel on day 3 and day 7 as the lower dosages of SAAP-148 and the earlier time points indicated similar results. The y-axis represents the log10 reduction (LR), which was calculated by subtracting the log number of surviving bacteria after treatment from the log number of bacteria in the negative control samples. The mean bacterial load of the negative control samples is indicated with a dotted line as the maximum LR. * indicates significant difference (*p<0.05).

**Table S1: Conversion of SAAP-148 dosages**

To compare the SAAP-148 dosages with 2% (wt/wt) Bactroban or Prontosan (positive controls), the amounts and concentrations of SAAP-148 in PBS (wt/v) or SAAP-148 in HM gel (wt/wt) are represented in the table.

| SAAP-148 (nmol) | Amount (µL) | SAAP-148 in PBS (%) |
| --- | --- | --- |
| 7.6 | 10 | 0.25 |
| 15 | 20 | 0.25 |
| 60 | 20 | 1 |
| 38 | 50 | 0.25 |
| 153 | 100 | 0.5 |
